# Real-world clinical impact of plasma cell-free DNA metagenomic next-generation sequencing assay

**DOI:** 10.1017/ice.2024.242

**Published:** 2025-05

**Authors:** Ishminder Kaur, Bennett Shaw, Ashrit Multani, Christine Pham, Sanchi Malhotra, Ethan Smith, Kristina Adachi, Paul Allyn, Zackary Bango, Omer Eugene Beaird, JR Caldera, Sukantha Chandrasekaran, Lynn Chan, Rabia Cheema, Sarah Daouk, Jaime Deville, Huan Vinh Dong, Austin Fan, Omai Garner, Pryce Gaynor, Hannah Gray, Aleksandr Gorin, Sowmya Kalava, Meganne Kanatani, Andrew Karnaze, Tawny Saleh, Yamini Sharma, Stacey Stauber, Moises Vargas, Monette Veral, Drew Winston, Lauren Yanagimoto-Ogawa, Grace Aldrovandi, Karin Nielsen-Saines, Trevon Fuller, Nicholas Jackson, Daniel Uslan, Joanna Schaenman, Tara Vijayan, Ashlyn Sakona, Shangxin Yang

**Affiliations:** 1 Division of Infectious Diseases, Department of Pediatrics, David Geffen School of Medicine, University of California, Los Angeles, CA, USA; 2 David Geffen School of Medicine at University of California, Los Angeles, CA, USA; 3 Division of Infectious Diseases, Department of Medicine, David Geffen School of Medicine, University of California, Los Angeles, CA, USA; 4 Department of Pharmaceutical Services, University of California, Los Angeles, CA, USA; 5 Department of Pathology and Laboratory Medicine, University of California, Los Angeles, CA, USA; 6 Department of Pediatrics, David Geffen School of Medicine, University of California, Los Angeles, CA, USA; 7 Institute of the Environment and Sustainability, University of California, Los Angeles, CA, USA; 8 Department of Medicine Statistics Core, University of California, Los Angeles, CA, USA

## Abstract

**Objective:**

To describe the real-world clinical impact of a commercially available plasma cell-free DNA metagenomic next-generation sequencing assay, the Karius test (KT).

**Methods:**

We retrospectively evaluated the clinical impact of KT by clinical panel adjudication. Descriptive statistics were used to study associations of diagnostic indications, host characteristics, and KT-generated microbiologic patterns with the clinical impact of KT. Multivariable logistic regression modeling was used to further characterize predictors of higher positive clinical impact.

**Results:**

We evaluated 1000 unique clinical cases of KT from 941 patients between January 1, 2017–August 31, 2023. The cohort included adult (70%) and pediatric (30%) patients. The overall clinical impact of KT was positive in 16%, negative in 2%, and no clinical impact in 82% of the cases. Among adult patients, multivariable logistic regression modeling showed that culture-negative endocarditis (OR 2.3; 95% CI, 1.11–4.53; *P* .022) and concern for fastidious/zoonotic/vector-borne pathogens (OR 2.1; 95% CI, 1.11–3.76; *P* .019) were associated with positive clinical impact of KT. Host immunocompromised status was not reliably associated with a positive clinical impact of KT (OR 1.03; 95% CI, 0.83–1.29; *P* .7806). No significant predictors of KT clinical impact were found in pediatric patients. Microbiologic result pattern was also a significant predictor of impact.

**Conclusions:**

Our study highlights that despite the positive clinical impact of KT in select situations, most testing results had no clinical impact. We also confirm diagnostic indications where KT may have the highest yield, thereby generating tools for diagnostic stewardship.

## Background

Prompt and accurate diagnosis of infectious diseases (ID) remains paramount to optimal management of hospitalized patients. Factors that limit the yield of standard microbiological tests include inherent delays of culture-based-diagnostic methods, pretreatment with antimicrobials, the fastidious nature of certain pathogens, challenges with invasive sampling, and insufficient clinical suspicion resulting in suboptimal selection of tests.^1^ Despite widespread commercial and hospital-level availability of polymerase chain reaction (PCR)-based tests, the need for advanced ID diagnostics remains. Plasma cell-free DNA (cfDNA) metagenomic next-generation sequencing assays (cf-mNGS) are gaining attention to bridge this gap.^2^

Currently, Karius Inc. (Redwood City, California) is the only reference laboratory that offers plasma cf-mNGS assay commercially under a CLIA license.^3^ Several reports evaluating the clinical utility of the Karius test (KT) have been published and certain variables are emerging as having potentially higher clinical impact but with differences across reports.^4^ Among diagnostic indications, data have shown that patients with culture-negative endocarditis, neutropenic fever, pneumonia in immunocompromised patients, and fever of unknown origin may benefit from plasma cf-mNGS testing.^5–8^ Among host characteristics, immunocompromised hosts seem to benefit from the use of plasma cf-mNGS testing.^9^ The clinical relevance of individual organisms and microbiological result patterns generated by KT have received limited investigation, even as an interpretation of polymicrobial results remains a diagnostic dilemma.^10–12^ Despite the cumulative published experience, the real-world clinical impact of KT in large patient populations is missing.

We examined the real-world clinical impact of KT in a large single-center retrospective cohort to define its clinical utility further stratified on diagnostic indications, host characteristics, and microbiologic result patterns.

## Methods

### Study design

This study was a retrospective review of 1000 unique cases of plasma cf-mNGS testing across adult and pediatric care settings at a large academic medical center in Los Angeles, California. Multiple KT performed on the same patient were included if they were separated by at least 6 months to ensure unique cases of plasma cf-mNGS testing were being reviewed.

These 1000 KT cases were assigned for clinical adjudication to 12 panels of investigators, with approximately 84 cases per panel. Each panel was comprised of 4 members: 2 ID specialists (at least 1 of them an attending physician), 1 clinical microbiologist, and an additional member (either an ID pharmacist, hospitalist attending physician, or medical student/resident). The clinical microbiologists were responsible for designation of the microbiological patterns of pathogens detected by KT into (A) 1 of 4 bacterial categories ((i) zoonotic/vector-borne, (ii) fastidious/slow-growing, (iii) 3+ bacterial genera detected, or (iv) other bacteria that does not fall in the previous 3 categories)), and/or (B) 1 of 3 fungal categories (mold, yeast, and dimorphic), and/or (C) viral, and/or (D) parasitic categories.^10^ Polymicrobial results could be assigned up to 3 microbiological patterns. Clinical adjudication was performed by the remaining 3 team members in each panel, whereby each member abstracted data for one-third of the assigned cases and reviewed the remaining cases abstracted by the other team members to ensure the validity of the abstracted data and allow for a minimum 3 assessments of each testing case.

Patient charts were reviewed to collect demographics, past medical history, clinical diagnosis, other microbiologic test results at the time of KT collection, and provider documentation pertaining to KT results. Patient comorbidities were assigned to discrete categories, including immunocompromised status, with further stratifications, or other comorbidities (Supplementary Table 1). Indications for KT were captured using 18 predefined categories, which included major clinical syndromes or concerns for fastidious organisms (Supplementary Table 2). Each patient could be assigned up to 3 comorbidity and 2 diagnostic indication categories. A positive clinical impact was defined as a KT result that led to (a) a new diagnosis not confirmed by standard microbiologic tests, (b) a new diagnosis made earlier than standard microbiologic tests, (c) additional diagnostic evaluation leading to a subsequent positive change in management, (d) avoidance of surgical/tissue biopsy, or (e) de-escalation/discontinuation of therapy. A negative clinical impact was predefined as a KT result that led to (a) unnecessary treatment, (b) unnecessary diagnostic evaluation, or (c) additional time spent in the hospital. No clinical impact was used to define cases where (a) KT results were not acted upon by clinical teams, (b) KT results confirmed a known diagnosis leading to no change in management, or (c) KT missed causative pathogen detected by standard microbiological tests. The indeterminate clinical impact was used where the clinical impact could not be determined from chart review.

Given the large number of investigators involved in clinical adjudication, we performed quality control assessments by assessing differences in the percentage of clinical impact designations by the panels. All but 2 panels were highly consistent in their clinical impact assessments. Our data validation reviews (performed by I.K., S.Y.) revealed that the differences in the 2 outlier panels stemmed from varied interpretations (result not acted upon, or indeterminate impact) of a KT test result that confirmed known diagnosis without change in management. To decrease this inconsistency without introducing further bias, we combined these 3 clinical impact categories (confirmed clinical suspicion without change in management, result not acted upon, could not determine impact) into no clinical impact.

Diagnostic stewardship of KT at our institution during the study period evolved from no oversight (before August 2018), to manual review of each order by microbiology postdoctoral staff to ensure approval by ID specialists (August 2018–February 2023), to computerized provider order entry (CPOE) restricted to ID physicians alone (after February 2023).

### Statistical approach

Descriptive statistics were used to describe the clinical impact of KT. Assessment of differences in clinical impact between adult and pediatric patient cohorts and during different phases of diagnostic stewardship at an institutional level were made using χ^2^ tests.

Further statistical analyses were performed to assess potential associations of clinical and microbiological variables with the positive clinical impact of KT. Pairwise univariable analysis of associations of diagnostic indications with positive clinical impact of KT were performed separately for the pediatric and adult patient cohort. Odds ratios (OR) were determined for each diagnostic indication, and statistical significance was determined by χ^2^ test or Fisher’s exact test, as appropriate. A similar analysis was done to determine the association of host comorbidities (separately for adult and pediatric cohorts) and KT microbiologic result pattern (combined for adult and pediatric cohorts) with the positive clinical impact of KT. A multivariable logistic regression model was constructed to further investigate the combined effects of the clinical variable(s) (diagnostic indications and host comorbidities) found to have statistically significant (*P* < .05) associations in the pairwise univariable analyses. All analyses were evaluated using a two-sided alpha level of 0.05 and conducted in R version 4.3.2.

This study was reviewed by the UCLA Human Research Protection Program and received an Institutional Review Board exemption.

## Results

### Demographics and KT result summary

**Figure 1.**
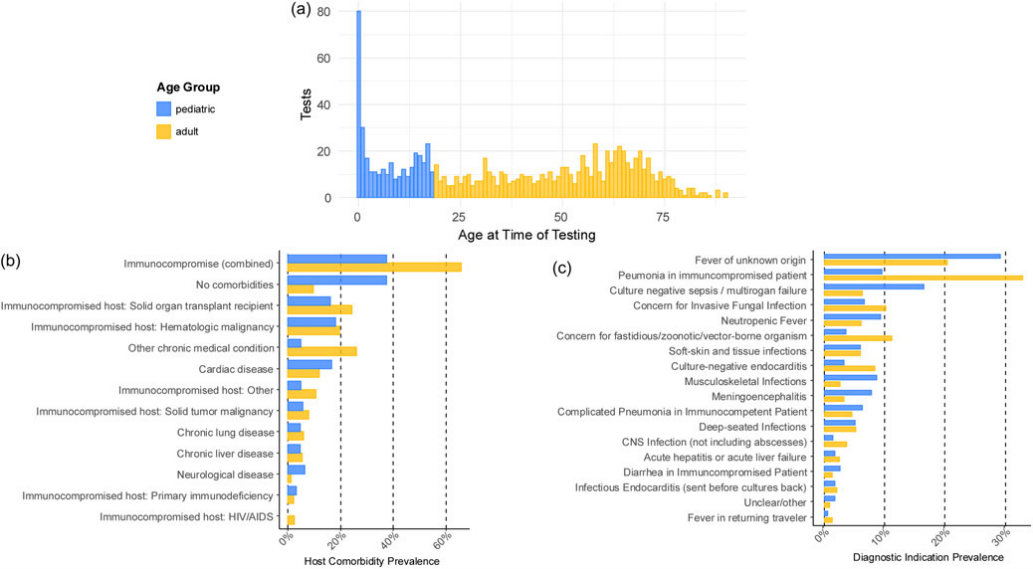
Demographics of the study population. Figure 1a, age distribution of the study cohort at the time of Karius testing; Figure 1b, distribution of comorbidities of the pediatric and adult cohort at the time of Karius testing; Figure 1c, distribution of diagnostic indications for Karius test in the pediatric and adult patient cohort.

The final cohort included 1000 cases from 941 individual patients tested using KT between January 1, 2017, through August 31, 2023. Of these patients, 57.9% were male. Of the 1000 unique cases, 299 tests were ordered for patients <18 years of age and 701 for patients 18 years and older (Figure 1a). Most adult patient cohorts had underlying comorbidities at the time of KT (627 of 701, 89.4%), compared with the pediatric patient cohort (194 of 299, 64.9%) (*P* < .01) (Figure 1b). The adult patient cohort had a higher percentage of immunocompromised patients (460 of 701, 65.6%), compared with the pediatric patient cohort (112 of 299, 37.4%) (*P* < .01) (Figure 1b). The most common diagnostic indications among pediatric patients were unexplained fevers, culture-negative sepsis/multiorgan failure, neutropenic fever, musculoskeletal infections, and meningoencephalitis. This contrasted with the adult patient population for whom pneumonia in immunocompromised patients, unexplained fevers, concern for fastidious/zoonotic/vector-borne organisms, concern for invasive fungal infection, followed by culture-negative endocarditis were the most common diagnostic indications (Figure 1c).

**Figure 2.**
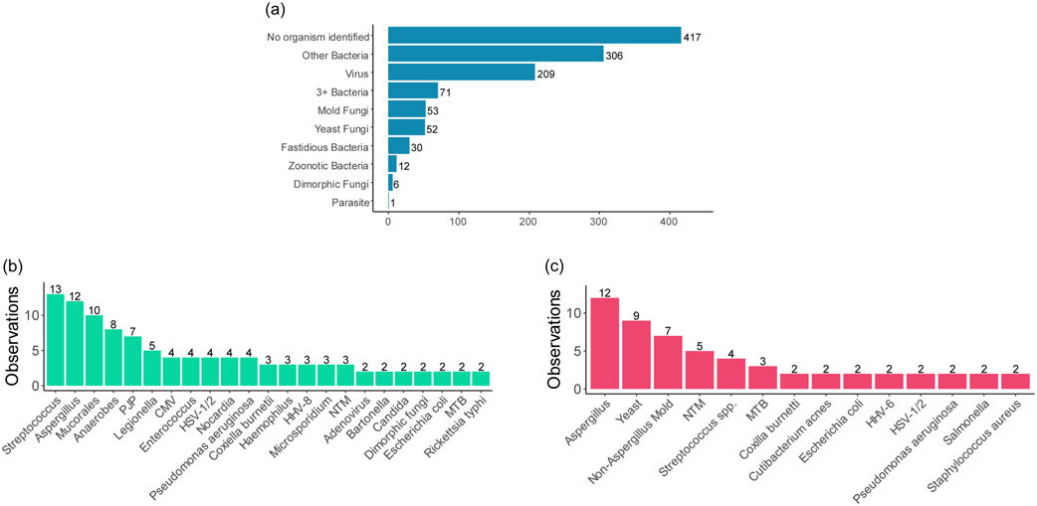
Microbiologic results of Karius testing. Figure 2a, Microbiologic result patterns of Karius test. Figure 2b, causative pathogens detected exclusively/earlier by Karius test in comparison with standard microbiological tests in more than one case. Figure 2c, causative pathogens missed by the Karius test in comparison with standard microbiological tests in more than one case.

At least one microorganism was detected by KT in 58.3% of cases, with the top 10 most frequently detected bacteria, fungi, and viruses shown in Supplementary Figure 1. The most common microbiologic result patterns of KT when organisms were identified were “other bacteria,” followed by viruses (Figure 2a).

### Clinical impact assessment

KT results were found to have no clinical impact in 82.2% (822 of 1000) of cases, predominantly due to the result not being acted upon by the clinical team (n = 565) (Table 1). KT results missed causative pathogens detected by standard diagnostic methods in 63 cases, with most of the missed organisms (36 of 63, 57.1%) being fungi or mycobacteria. A fungal etiology was missed by KT in 28 cases (12 of which were *Aspergillus* spp.), and mycobacteria were missed in 8 cases (including 3 cases of *Mycobacterium tuberculosis*, all diagnosed by tissue biopsy). Frequently missed causative pathogens are shown in Figure 2c. The standard microbiologic tests and specimen sources that identified the frequently missed pathogens by KT are shown in Supplementary Table 3, with sites of infection summarized in a footnote. In cases where KT missed identifying the causative pathogen, it either generated results with other nonrelevant microorganisms (n = 26) or no microorganisms (n = 37).

**Table 1.** Summary of clinical impact of Karius results (total N = 1000)

Impact	Impact rationale	Cases, N
None (n=822)	Result not acted upon by clinical team	565
	Karius result confirmed a known diagnosis leading to no change in management	130
	Could not determine the clinical impact from chart review	64
	Karius missed the causative pathogen detected by standard microbiologic methods	63
Positive (n=162)	New diagnosis not confirmed by standard diagnostic methods	69
	New diagnosis made earlier by Karius result than standard microbiological methods	34
	Karius result led to additional diagnostic investigations that led to positive changes in management	12
	Karius test enabled avoidance of invasive tissue or surgical biopsy	0
	Karius result enabled de-escalation or discontinuation of therapy	47
Negative (n=16)	Karius result led to additional unnecessary diagnostic investigations	11
	Karius result led to unnecessary treatment per the infectious diseases team involved	4
	Karius result led to additional time spent in the emergency department or prolonged hospital length of stay	1

Positive impact was found in 162 cases (16.2%), largely due to a new diagnosis not confirmed by (n = 69) or made earlier (n = 34) than standard microbiologic tests (Table 1). The most frequent causative pathogens detected exclusively or earlier by KT are shown in Figure 2b and include *Streptococcus* spp. (including *S. pneumoniae* and *S. pyogenes*), *Aspergillus* spp., *Mucorales*, anaerobes, *Pneumocystis jirovecii*, and *Legionella.*

Negative impact was found in only 16 cases (1.6%). This was largely (n = 11) due to KT results leading to additional unnecessary diagnostic investigations, including quantitative PCRs for viruses found on KT or imaging to evaluate for endocarditis or occult intra-abdominal infection following detection of oral flora or enteric anaerobes by KT.

### Association of clinical impact with clinical variables

KT had a positive clinical impact in 43 of 299 pediatric patients (14.4%) and 120 of 701 adult patients (17%) (Supplementary Figure 2a). These differences were not statistically significant (χ^2^ test, *P* = .17).

We observed a trend toward increasing positive impact rate with the implementation of each phase of diagnostic stewardship from 5.6% (1 of 18) at baseline to 15.9% (116 of 729) with a manual review of ID approval by microbiology postdoctoral fellows and to 17.8% (45 of 253) with CPOE restricted to ID providers alone (Supplementary Figure 2b). These differences were not statistically significant (Fisher’s exact test *P* = .50).

Pairwise univariable analysis of diagnostic indications with positive clinical impact of KT showed no significant association in the pediatric cohort, while culture-negative endocarditis and concern for fastidious/zoonotic/vector-borne pathogens showed significant positive association in the adult cohort (*P* values of .033 and .032, respectively) (Table 2). Meningoencephalitis was negatively associated with the clinical impact of KT in adult patients (*P*-value of .048) (Table 2). Exclusive or earlier pathogen detection by KT done for diagnostic indications that either trended toward or were associated with higher positive clinical impact are shown in Supplementary Table 4.

**Table 2.** Pairwise univariable analysis of diagnostic indication with the positive clinical impact of Karius testing among pediatric and adult patients

	Pediatrics (n=299)			Adults (n=701)		
Diagnostic indication	Positive impact with indication	Positive impact without indication	*P*-value	Positive impact with indication	Positive impact without indication	***P*-value**
Acute liver failure or hepatitis	3/17 (17.6%)	40/282 (14.2%)	.969	0/6 (0%)	119/695 (17.1%)	.571
CNS infection including lesions (not abscess)	1/4 (25%)	42/295 (14.2%)	1.000	2/26 (7.7%)	117/675 (17.3%)	.308
Complicated pneumonia in immunocompetent patients	6/20 (30%)	37/279 (13.3%)	.083	6/32 (18.75%)	113/669 (16.9%)	.974
Concern for fastidious/zoonotic/vector-borne pathogens	4/11 (36.4%)	39/288 (13.5%)	.093	20/76 (26.3%)	99/625 (15.8%)	.033^a^
Culture-negative endocarditis	1/10 (10%)	42/289 (14.5%)	1.000	16/57 (28.1%)	103/644 (16.0%)	.032^a^
Culture-negative sepsis or multiorgan failure	7/51 (13.7%)	36/248 (14.5%)	1.000	10/46 (21.7%)	109/655 (16.6%)	.492
Deep-seated infections including abscesses	2/16 (12.5%)	41/283 (14.5%)	1.000	10/36 (27.8%)	109/665 (16.4%)	.122
Diarrhea in immunocompromised patients	0/7 (0%)	43/292 (14.7%)	1.000	4/11 (36.4%)	115/690 (16.7%)	.186
Unexplained fevers	13/88 (14.8%)	30/211 (14.2%)	1.000	21/145 (14.5%)	98/556 (17.6%)	.439
Fever or illness in a returning traveler	0/1 (0%)	43/288 (14.4%)	1.000	2/10 (20%)	117/691 (16.9%)	1.000
Infective endocarditis (sent before cultures back)	1/4 (25%)	42/285 (14.2%)	1.000	2/16 (12.5%)	117/685 (17.1%)	.884
Invasive fungal infection	5/21 (23.8%)	38/278 (13.7%)	.340	7/69 (10.1%)	112/632 (17.7%)	.155
Meningoencephalitis	5/24 (20.8%)	38/275 (13.8%)	.525	0/24 (0%)	119/677 (17.6%)	.048^a^
Musculoskeletal infections	4/24 (16%)	39/274 (14.2%)	1.000	3/22 (13.6%)	116/679 (17.1%)	.892
Neutropenic fever	1/21 (4.8%)	42/278 (15.1%)	.327	10/51 (19.6%)	109/650 (16.8%)	.744
Pneumonia in immunocompromised patients	3/25 (12%)	40/274 (14.6%)	.955	43/226 (19.0%)	76/475 (16.0%)	.373
Skin and soft tissue infections	3/19 (15.8%)	40/280 (14.2%)	1.000	6/41 (14.6%)	113/660 (17.1%)	.844
Unclear/other	0/6 (0%)	43/293 (14.7%)	.670	0/3 (0%)	119/698 (17.0%)	.989

Note. CNS, central nervous system.

a *P* < .05.

Pairwise univariable analysis of host comorbidities showed no association with positive clinical impact in the pediatric or adult patient cohorts (Supplementary Table 5).

**Multivariable regression model** was constructed only for the adult patient cohort with the input of culture-negative endocarditis, concern for fastidious/zoonotic/vector-borne pathogens, central nervous system infections (lesions or encephalitis combined based on the similar negative signal in pairwise univariable analysis), deep-seated infections, invasive fungal infection, pneumonia regardless of immunocompromised status, and host immunocompromised status. The host immunocompromised status was selected based on prior published data of association with higher KT clinical impact. Pneumonia cases were combined regardless of host immune status due to concerns of collinearity with the incorporation of immunocompromise as a variable in the model construction. Multivariable analysis showed that among the adult patient cohort, culture-negative endocarditis (OR 2.27; 95% CI, 1.11–4.53; *P* .022) and concern for fastidious/zoonotic/vector-borne pathogens (OR 2.07; 95% CI, 1.11–3.76; *P* .019) were associated with higher positive clinical impact of KT (Table 3). Host immunocompromised status was not significantly associated with the positive clinical impact of KT (OR 1.03; 95% CI, 0.83–1.29; *P* .7806) (Table 3).

**Table 3.** Results of multivariable analysis of specific diagnostic indications and comorbidities (selected based on results of pairwise univariable analysis or known significance in prior studies) to determine association with positive clinical impact of an adult patient cohort

Parameter	Significance level (*P*-value)	Odds ratio (95% confidence interval)
Concern for fastidious/zoonotic/vector-borne pathogens	.0187^a^	2.07 (1.11–3.76)
Culture-negative endocarditis	.0218^a^	2.27 (1.11–4.53)
Deep-seated infection	.0648	1.18 (0.98–1.40)
CNS infection (lesion or meningoencephalitis)	.0690	0.71 (0.44–0.97)
Pneumonia	.1072	1.22 (0.95–1.56)
Invasive fungal infection	.2167	0.59 (0.23–1.28)
Host immunocompromised status	.7806	1.03 (0.83–1.29)
Unexplained fevers	.8202	0.96 (0.52–1.64)

Note. CNS, central nervous system.

a *P*-value < .05.

### Association of clinical impact with microbiologic patterns of KT result

Pairwise univariable analysis revealed that several microbiologic result patterns of KT were associated with a higher positive clinical impact, namely zoonotic/vector-borne bacteria, followed by fastidious/slow-growing bacteria, molds, and yeasts (Table 4). KT results designated as other bacteria, viruses, and 3+ bacterial genera were associated with lower positive clinical impact.

**Table 4.** Pairwise univariable analysis of microbiologic result patterns of Karius testing with its positive clinical impact (results represent the combined pediatric and adult patient cohort)

Microbiologic pattern	Positive impact with the pattern	Positive impact without the pattern	Significance level (*P*-value)
Zoonotic/vector-borne bacteria	10/12 (83.3%)	127/571 (22.2%)	<.001^a^
Fastidious/slow-growing bacteria	18/30 (60.0%)	119/553 (21.5%)	<.001^a^
Molds	24/53 (45.3%)	113/530 (21.3%)	<.001^a^
Yeasts	23/52 (44.2%)	114/531 (21.5%)	<.001^a^
Dimorphic fungi	2/6 (33.3%)	135/577 (23.4%)	.931
Virus	39/209 (18.7%)	98/374 (26.2%)	.05
Other bacteria	56/316 (18.3%)	81/277 (29.2%)	.003^a^
3+ bacterial genera	11/76 (14.5%)	135/577 (23.4%)	.122

a *P* < .05.

## Discussion

Our study shows that in a large academic center with a high volume of KT testing, most ordered KT had no clinical impact (82.2%), while smaller percentages had a positive (16.2%) or a negative (1.6%) clinical impact. We also found a trend toward improving clinical impact over time in parallel to more ID provider oversight. Multivariable logistic regression modeling found that the highest positive clinical impact of KT in adult patients is seen when performed for select diagnostic indications, namely, culture-negative endocarditis, and concern for fastidious/zoonotic/vector-borne pathogens.

Our findings are supported by the role of KT in culture-negative endocarditis and Q fever in recent studies.^13–15^ We did not find an association of positive clinical impact with unexplained fevers, but this cohort was not strictly defined to include patients with fever of unknown etiology and more often included patients with unexplained hospital-onset fevers. Host immunocompromised status is often described as an important consideration in predicting the clinical impact of plasma cf-mNGS testing.^9^ A recent pediatric study showed a limited impact of plasma cf-mNGS testing among immunocompromised children.^16^ Our study suggests that while KT may increase the detection of clinically relevant pathogens, the positive clinical impact of KT is modulated more by the type of infection and the nature of suspected pathogens as opposed to the host immune status. We also did not find that pneumonia in immunocompromised patients was a significant predictor of a higher positive clinical impact of KT. A recent prospective study of immunocompromised patients with pneumonia showed that KT provided an additive diagnostic value of 17.4% in 121 patients where standard methods failed to identify an etiology.^7^ This could be in part that, fungal and mycobacterial pathogens, even though highly impactful when KT detects them, also represented most of the “missed” causative pathogens by KT. Several of these diagnoses were made in our patient cohort by pursuing invasive tissue specimens, a finding similar to other investigations.^17^

We found meningoencephalitis as an indication was inversely associated with positive clinical impact (0%, 0 of 24) in adult patients, but not in pediatric patients (21%, 5 of 24). No other literature has demonstrated the diagnostic utility of using plasma cf-mNGS for meningoencephalitis in adults. Instead, direct cerebrospinal fluid mNGS has shown promise in improving the diagnostic yields for meningoencephalitis.^18^ In pediatric patients, however, one small study showed plasma cf-mNGS may provide some added value as an adjunct to standard testing for meningitis.^19^

Our study also found that false-negative KT results were seen in 63 clinical cases. This confirms the known problematic sensitivity and specificity of KT.^4,9^ KT implementation at an institutional level should be accompanied by provider education highlighting that KT does not have the sensitivity of dedicated single microbiologic tests to detect specific targets and should not be relied upon as the sole modality to rule out an infectious etiology in the context of high clinical suspicion. Increased uptake of dedicated testing, such as targeted 16S rRNA for infective endocarditis or a targeted PCR for zoonotic pathogens has the potential to decrease the positive impact of KT.^20^

Our study also lends itself to the comparison of the overall clinical utility of KT testing between adult and pediatric patient populations. Complicated pneumonia in immunocompetent patients and concern for fastidious/zoonotic/vector-borne pathogens trended toward association with positive clinical impact among pediatric patients, but results did not reach significance, likely in part due to smaller sample size. Other investigators have shown positive impact in pediatric patients with complicated pneumonia.^21^ We also noted that a substantial number of KT were sent among infants. This warrants further study as the role of plasma cf-mNGS has received limited investigation in this cohort.^22^

Our study has several limitations, including the retrospective and heterogeneous nature of the study cohort. The timing of the KT in the clinical course was up to the discretion of the ordering providers with variability between cases and could have affected its clinical impact. The large number of investigators could be seen as a limitation, but the clinical adjudication was highly aligned and data validation revealed discrepancies that were resolved prior to analysis. The statistical analysis of predictive factors for the positive impact of KT was performed by comparing it to clinically adjudicated diagnostic results in a real-world clinical setting. The standard microbiological testing available to the providers includes a comprehensive repertoire of tests as would be expected for a large academic health system. The results of the multivariate regression modeling should be interpreted in the light of the limitations of the study design and without a priori definition of power needed for such analysis. Further, the real-world clinical impact of the KT will likely be different for centers that have had more intensive diagnostic stewardship oversight of the test upstream—before broad application by front line providers. Lastly, this study was not designed to be a comparison of the clinical performance of KT compared to the standard microbiologic tests because such work has been previously published.^4,17,23–25^

Importantly, we showed the lack of clinical impact of plasma cf-mNGS testing with the trend toward improved impact after implementing increased oversight. This highlights the complex role for plasma cf-mNGS testing and the importance of optimizing diagnostic stewardship upfront. We have hopefully generated useful information for diagnostic stewardship of plasma cf-mNGS testing. These data are important as the cost-effectiveness of KT is being explored and given the significant cost of KT.^26^ Our findings suggest that successful implementation of KT in any institution requires a nuanced, multidisciplinary approach involving ID providers and clinical microbiologists to ensure optimal patient selection and ongoing provider education to aid in test result interpretation. Future prospective studies are needed to better define the role of KT.

## Supporting information

Kaur et al. supplementary material Kaur et al. supplementary material

## Supplementary material

The supplementary material for this article can be found at https://doi.org/10.1017/ice.2024.242.
