# Supplementary material for: Real-world clinical impact of plasma cell-free DNA metagenomic next-generation sequencing assay
Source: Infect Control Hosp Epidemiol. 2025 Jan 30;46(5):504–11. doi: 10.1017/ice.2024.242 (PMC12034450; doi:10.1017/ice.2024.242)

**Supplementary Table 1.** Comorbidities Categorization for analysis of Karius Testing Impact

| **Comorbidities Categorization** |
| --- |
| 1. Immunocompromised host: Solid tumor malignancy |
| 1. Immunocompromised host: Hematologic malignancy (including HCT and/or CAR-T recipient) |
| 1. Immunocompromised host: Solid organ transplant recipient |
| 1. Immunocompromised host: Primary immunodeficiency disorder (e.g., severe combined immunodeficiency, DiGeorge syndrome, Wiskott-Aldrich syndrome, common variable immunodeficiency disease, etc.) |
| 1. Immunocompromised host: HIV/AIDS |
| 1. Immunocompromised host: Other, including rheumatologic/autoimmune conditions and on immunosuppressive therapy, asplenia, other immunosuppressive therapy (steroids, immunomodulatory biologic agents, etc.) |
| 1. Cardiac diseases: Prosthetic valves or materials, complex congenital cardiac disease, cardiomyopathy |
| 1. Chronic liver diseases: Cirrhosis, Non-alcoholic fatty liver disease, Alcoholic liver disease, Autoimmune hepatitis |
| 1. Chronic lung diseases: Cystic fibrosis, bronchiectasis, COPD, interstitial lung disease, pulmonary hypertension, bronchopulmonary dysplasia, asthma |
| 1. Neurological diseases: VP shunt, congenital brain malformations, cerebrovascular disease, epilepsy, cerebral palsy |
| 1. Other chronic medical conditions (chronic kidney disease, end stage renal disease, dialysis dependent, diabetes mellitus, hypertension, sickle cell disease) |
| 1. No comorbidities |

**Supplementary Table 2.** Diagnostic Criteria for Karius Testing

| **Diagnostic Criteria for Karius Testing** |
| --- |
| 1. Acute liver failure or hepatitis |
| 1. CNS infection including lesions (not abscess) (discrete lesions, not abscess, not meningoencephalitis) |
| 1. Complicated pneumonia in immunocompetent patients |
| 1. Concern for fastidious organisms (like Bartonella, Rickettsia, Q fever) or zoonotic/vector-borne pathogens |
| 1. Culture-negative Endocarditis |
| 1. Culture negative sepsis or Multiorgan failure |
| 1. Deep seated infections including abscesses (liver abscess, brain abscess, intra-abdominal abscess, or endophthalmitis) |
| 1. Diarrhea in immunocompromised patient |
| 1. Unexplained fevers |
| 1. Fever or illness in a returning traveler |
| 1. Infective Endocarditis (test sent before blood cultures back) |
| 1. Invasive Fungal Infection |
| 1. Meningoencephalitis |
| 1. Musculoskeletal Infections, including septic arthritis |
| 1. Neutropenic fever |
| 1. Pneumonia in immunocompromised patient |
| 1. Skin and soft tissue infections, including rashes |
| 1. Unclear/other |

**Supplementary Table 3.** Frequently missed pathogens by KT (missed in at least 3 episodes) and the standard microbiologic tests alongside the specimen source that identified the pathogen.

| **Pathogen Missed by KT** | **Microbiologic Tests and Specimen Source that Identified the Pathogen Missed by KT** |
| --- | --- |
| *Aspergillus* spp. (12 episodes) | Culture in 10 episodes (source: skin and soft tissue n=2, brain tissue n=1, lung tissue n=1, upper respiratory n=2, BAL n=4), broad range PCR in one episode (source: BAL n=1), aspergillus- EIA in one episode (source: BAL n=1) |
| Yeast^a^ (9 episodes) | Culture in all 9 episodes (source: tissue n=1, blood n=2, cerebrospinal fluid n=2, vitreous fluid n=1, blood and tissue n=1, esophageal tissue n=1, blood + pleural fluid + BAL n=1) |
| Non-*Aspergillus* Mold^b^ (7 episodes) | Culture in 6 episodes (source: peritoneal n=1, bone and soft tissue n=1, blood n=1, upper respiratory n=1, blood and invasive tissue n=1, lung tissue n=1), tissue pathology and broad range PCR in one episode (source: tissue n=1) |
| Non-tuberculous mycobacteria^c^ (5 episodes) | Culture in all 5 episodes (source: blood n=1, lung tissue n=1, BAL n=1, soft tissue n=2) |
| Streptococcus spp.^d^ (4 episodes) | Culture in all 4 episodes (source: brain tissue n=1, blood n=1, lymph node n=1, neck tissue n=1) |
| Mycobacterium tuberculosis (3 episodes) | Culture in one episode (source: pleural fluid and bone aspirate n=1), tissue pathology and later broad range PCR (source: lymph node n=1), serology and pathology in one episode (source: blood for serology and tissue for pathology n=1) |

BAL, bronchoalveolar lavage

Primary sites of infection: Skin and soft tissue (4), Lung (19), Brain (6), Bone and/joint (2), ocular (1), Esophagus (1), Mediastinum (1), Paranasal sinuses (1), Intra-abdominal (1), Blood stream infection (2), Lymph node (1), Deep neck space (1)

^a^Yeast included *Cryptococcus neoformans* (1), *Candida metapsilosis* (1), *Candida parapsilosis* (1), *Candida albicans* (1), *Candida glabrata* (1), *Candida* spp (1), *Blastobotrys raffinosifermentans* (1), *Naganisha* (1), *Malassezia furfur* (1)

^b^Non-aspergillus mold included *Alternaria* (2), *Arthrographis kalrae* (1)*,* *Fusarium* (1), *Trichosporon asahii* (1), *Scedosporium* (1), and mold not further identified

^C^Non-tuberculous mycobacteria included *M. fortuitum* (1), Mycobacterium avium complex (2), *M. chimaera* (1), *M. haemophilum* (1)

^d^Streptococcus spp. included *S. anginosus* (2), *S. viridans* (1), *S. pyogenes* (1)

**Supplementary Table 4.** Exclusive or earlier detection of pathogens by KT done for diagnostic indications that either trended towards or were significantly associated with positive clinical impact.

| **Diagnostic Category** | **Microbiologic Pattern** | **Pathogen Detected by Karius Test (episodes # if > 1)** |
| --- | --- | --- |
| Culture-negative endocarditis | Zoonotic/vector-borne bacterial pathogens | *Bartonella quintana* (n=3), *Coxiella burnetii* (n=2), *Streptobacillus moniliformis* |
|  | Mold | *Aspergillus fumigatus*, *Aspergillus nidulans* |
|  | Bacteria, other | *Staphylococcus aureus*, *Streptococcus pasteurianus*, *Enterococcus faecalis*, *Pseudomonas aeruginosa, Haemophilus parainfluenzae* |
| Concern for fastidious organisms or zoonotic/vector-borne pathogens | Bacterial- fastidious and slow growing | *Mycobacterium tuberculosis* complex*, Streptococcus pneumoniae, Nocardia cyriacigeorgica, Legionella pneumophila* |
|  | Zoonotic/vector-borne bacterial pathogens | *Bartonella quintana* |
|  | Bacteria, other | *Streptococcus sanguinis, Streptococcus oralis, Helicobacter bilis* |
|  | Mold | *Aspergillus tubingensis* |
|  | Dimorphic fungi | *Histoplasma capsulatum* |
|  | Yeast | *Encephalitozoon cuniculi* |
|  | Virus | Human adenovirus C |
|  | 3+ Bacteria | Anaerobes |
| Deep seated abscesses | Fastidious/slow-growing bacterial pathogens | *Mycobacterium abscessus, Mycobacterium tuberculosis* complex |
|  | Bacteria, other | Anaerobes *(n=4), Streptococcus (n=2), Enterococcus (n=2)* |
| Complicated pneumonia in immunocompetent patients | Fastidious/slow-growing bacterial pathogens | *Legionella* (n=2), *Streptococcus pneumoniae* (n=2) |
|  | Bacteria, other | Anaerobes (n=3), *Streptococcus pyogenes* |
|  | Virus | HSV-2 |
| Pneumonia in immunocompromised patients | Fastidious/slow-growing bacterial pathogens | *Nocardia* (n=3), *Legionella* (n=2), *Mycobacterium kansasii* |
|  | Bacteria, other | Anaerobes (n=2) |
|  | Mold | *Aspergillus* (n=8), *Mucorales* (n=8) |
|  | Dimorphic fungi | *Coccidioides immitis* |
|  | Yeast | *Pneumocystis jirovecii (n=7)* |
| Culture-negative sepsis | Zoonotic/vector-borne bacterial pathogens | *Rickettsia typh*i (n=2) |
|  | Bacteria, other | *Escherichia coli*, *Pseudomonas aeruginosa* |
|  | Yeast | *Pneumocystis jirovecii* |
|  | Virus | HSV-1/2 (n=2), adenovirus, CMV |

**Supplementary Table 5.** Bivariate analysis of host comorbidities with positive clinical impact of Karius testing in pediatric and adult patients.

|  | **Pediatrics (n=299)** | | | **Adults (n=701)** | | |
| --- | --- | --- | --- | --- | --- | --- |
| **Host Comorbidity at the time of Karius testing** | **Positive impact with comorbidity** | **Positive impact without comorbidity** | **p-value** | **Positive impact with comorbidity** | **Positive impact without comorbidity** | **p-value** |
| Host immunocompromised status (combined) | 15/97 (15%) | 28/159 (18%) | 0.836 | 77/383 (20%) | 42/199 (21%) | 0.901 |
| Solid organ transplant recipient | 10/30 (33.3%) | 33/226 (15%) | 0.070 | 26/152 (17%) | 93/430 (22%) | 0.390 |
| Hematologic malignancy | 5/38 (13%) | 38/218 (17%) | 0.748 | 27/122 (22%) | 92/460 (20%) | 0.767 |
| Solid tumor malignancy | 1/14 (7%) | 42/242 (17%) | 0.620 | 8/49 (16%) | 111/533 (21%) | 0.665 |
| Primary Immunodeficiency | 0/10 (0) | 43/246 (17%) | 0.390 | 1/15 (7%) | 118/567 (21%) | 0.410 |
| HIV/AIDS | N/A | | | 3/14 (21%) | 116/568 (20%) | 1.000 |
| Other immunocompromise | 1/12 (8%) | 42/244 (17%) | 0.620 | 16/60 (27%) | 103/522 (20%) | 0.400 |
| Chronic liver disease | 3/9 (33.3%) | 40/247 (16%) | 0.516 | 10/30 (33%) | 109/552 (20%) | 0.240 |
| Chronic lung disease | 2/11 (18%) | 41/245 (17%) | 1.000 | 4/38 (11%) | 115/544 (21%) | 0.265 |
| Cardiac disease | 4/42 (9%) | 39/214 (18%) | 0.334 | 16/71 (23%) | 103/511 (20%) | 0.883 |
| Neurological Disease | 4/14 (29%) | 39/242 (16%) | 0.528 | 0/9 (0) | 119/573 (21%) | 0.358 |
| No comorbidities | 16/89 (0.18) | 27/167 (16%) | 0.890 | 14/60 (23%) | 105/522 (23%) | 0.759 |

*p-value <0.05

**Supplementary Figure 1.** Top 10 most frequently detected bacteria, fungi and viruses by Karius Test

**Supplementary Figure 2.** Clinical impact of Karius Test based on (a) age, and (b) institutional diagnostic stewardship measures in place at time of Karius testing.

Impact

Positive

Negative

Inconsequential

1. b)


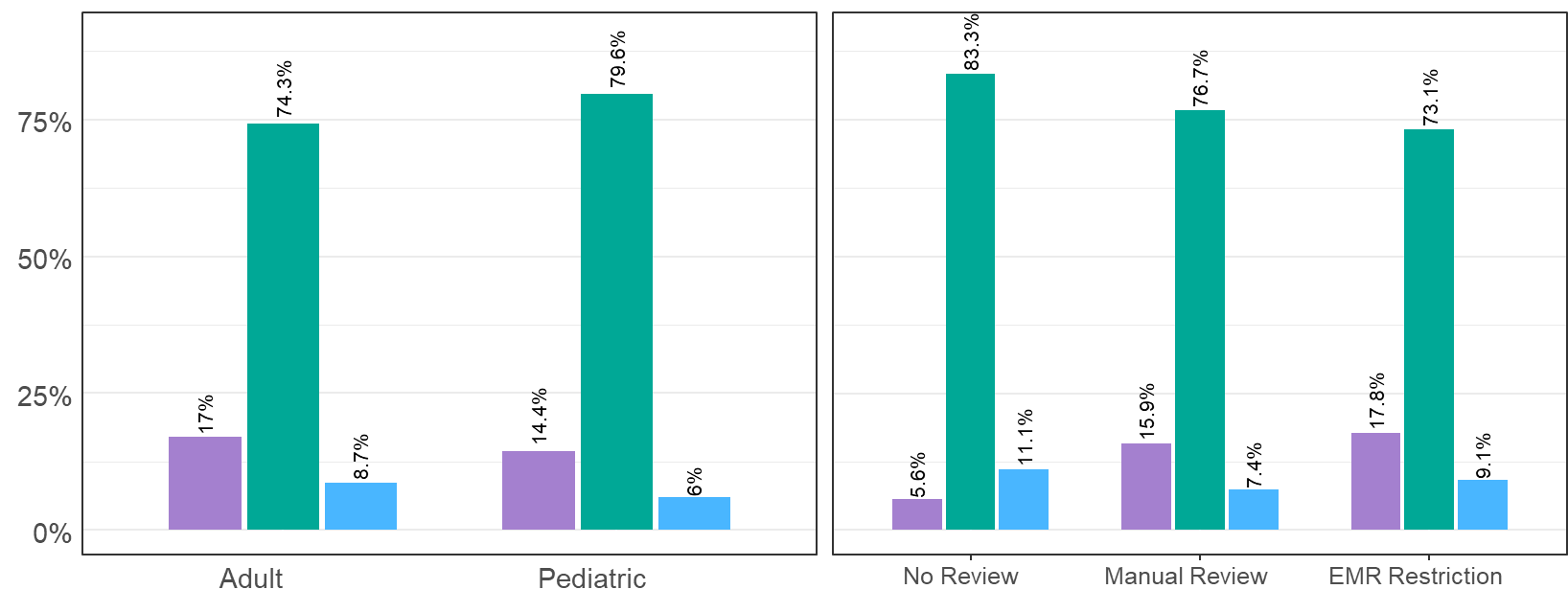

Supplement: Kaur et al. supplementary material [file S0899823X24002423sup001.docx]
